# Prenatal repair of myelomeningocele is associated with lower need for long-term feeding support

**DOI:** 10.1038/s41372-025-02356-4

**Published:** 2025-07-23

**Authors:** Jennifer Healy, Chunyan Liu, Shelley Ehrlich, Foong-Yen Lim, Jose L. Peiro, Beth Haberman, Charles B. Stevenson, Stefanie Riddle

**Affiliations:** 1 Division of Neonatology and Department of Pediatrics, Cincinnati Children’s Hospital Medical Center and University of Cincinnati College of Medicine, Cincinnati, OH USA; 2 Division of Biostatistics and Epidemiology, Cincinnati Children’s Hospital Medical Center, Cincinnati, OH USA; 3 Department of Environmental and Public Health Sciences, University of Cincinnati College of Medicine, Cincinnati, OH USA; 4 Division of Pediatric General and Thoracic Surgery, Cincinnati Children’s Hospital Medical Center, Cincinnati, OH USA; 5 Cincinnati Children’s Fetal Care Center, Cincinnati, OH USA; 6 Division of Pediatric Neurosurgery, Cincinnati Children’s Hospital Medical Center, Cincinnati, OH USA

**Keywords:** Outcomes research, Paediatrics

## Abstract

**Objective:**

Infants with myelomeningocele (MMC) are at risk of brainstem dysfunction secondary to symptomatic Chiari II malformation with hindbrain herniation (HH), which can manifest as feeding difficulties including aspiration and dysphagia. This study aims to investigate whether prenatal repair of MMC is associated with improved feeding outcomes compared to postnatal repair.

**Study design:**

Retrospective observational study of 208 infants with MMC, 105 repaired prenatally and 103 repaired postnatally, from January 2011 to July 2022. Primary outcome was feeding tube at discharge and longitudinally through 12 months corrected gestational age (CGA).

**Results:**

9.5% of infants repaired prenatally and 13.6% repaired postnatally required feeding tube at discharge (*p* = 0.3585). By 53 weeks CGA, the prenatal repair group had decreased odds of requiring feeding tube (0.325 [95% CI 0.121, 0.872]).

**Conclusion:**

Prenatal MMC repair was associated with decreased need for long-term feeding support, suggesting a potential functional benefit of prenatal repair related to reversal of HH.

## Introduction

Spina bifida is a common congenital anomaly occurring in 3.6 per 10,000 live births in the United States [1]. Myelomeningocele (MMC) is the most common and severe form of spina bifida, and survivors have high rates of disability including lower extremity paralysis and bowel and bladder dysfunction [2, 3]. Infants with MMC have associated Arnold-Chiari II malformation (CIIM) with varying degrees of hindbrain herniation (HH), which is characterized by caudal descent of the medulla, fourth ventricle, and cerebellar vermis into the cervical spinal canal. Approximately 80-90% of these infants will develop progressive hydrocephalus requiring surgical cerebrospinal fluid (CSF) diversion, such as placement of a ventriculoperitoneal (VP) shunt [3, 4].

Prenatal MMC repair was developed in an attempt to alleviate many of these morbidities [5]. In a multi-site randomized trial designed to study the safety and efficacy of prenatal MMC repair, the landmark Management of Myelomeningocele Study (MOMS) found that it resulted in lower need for VP shunt placement by 12 months of life, improved composite scores in mental development and motor function at 30 months, and a high rate of reversal of HH when compared to standard postnatal repair [6]. Subsequent studies have confirmed this finding, with anatomic HH reversal rates as high as 70-96% [7–9]. Despite these positive outcomes, prenatal MMC repair carries significant risks including premature birth and obstetric complications [10, 11]. The risks of premature birth are well known, and while only 13% of patients in the MOMS cohort delivered at less than 30 weeks gestation, the average gestational age was 34.1 weeks in the prenatal repair group. The developmental immaturity of the late preterm infant includes a frequent delay in the acquisition and progression of oral feeding skills, and many infants will require short-term feeding support until that skill and maturity is acquired [12].

Infants with MMC, regardless of repair strategy, also frequently face environmental and medical barriers to feeding. These may include time spent *nil per os* (NPO) due to surgery and need for intubation around the time of repair, as well as positioning restrictions related to post-operative recovery and wound care needs. While this can affect bottle feeding, it may present even greater challenges to breastfeeding, as well as other bonding interactions such as holding and skin-to-skin (STS) contact [13, 14].

As many as one-third of patients with MMC and CIIM will develop associated symptoms of caudal brainstem dysfunction. In infants, this can manifest as symptoms such as apnea, stridor, weak cry, vocal cord paralysis, and feeding difficulties, and severe symptoms can be life threatening [3, 4, 11]. Feeding difficulties can include aspiration, neurogenic dysphagia and swallowing dysfunction, choking, long feeding times, and reflux, which can necessitate use of a feeding tube. In a small case series analyzing infants with symptomatic CIIM, swallowing dysfunction was the most prevalent symptom, occurring in 21 of 32 patients (66%). These symptoms can affect quality of life and have downstream effects including failure to thrive, aspiration pneumonia and nutritional deficiencies [15–19].

By reversing anatomic HH, prenatal repair may improve symptoms of brainstem dysfunction, such as feeding difficulties. Conversely, prematurity related to prenatal repair may counteract these benefits, as premature infants also face feeding challenges related to developmental immaturity and frequently require feeding tubes [12]. In a survey sent to parents of prenatally repaired infants, parent-reported neurogenic dysphagia was present in 23%, but only 2% had severe symptoms including aspiration and need for gastrostomy tube (GT) [20]. A study comparing brainstem dysfunction in prenatally versus postnatally repaired infants found that no infants repaired prenatally had symptomatic brainstem dysfunction, compared to 25% of those repaired postnatally. Moreover, neurogenic dysphagia requiring GT was the most common manifestation of brainstem dysfunction in the postnatal repair group [21]. Despite evidence of the prevalence of swallowing dysfunction in infants with symptomatic CIIM, no studies have directly compared feeding outcomes between prenatal and postnatal repair strategies. In this study, we aim to determine whether there are differences in need for feeding support between infants undergoing prenatal and postnatal MMC repair, which may suggest that prenatal repair is associated with decreased CIIM-related feeding dysfunction. While major benefits of prenatal repair have been demonstrated, this study can provide additional information about an important outcome to aid in prenatal counseling regarding repair type as well as placement and management of feeding tubes in these infants.

## Methods

The Cincinnati Children’s Fetal Care Center database and electronic medical record were used to identify patients for this retrospective study. Infants born between January 2011 and July 2022 with a diagnosis of open neural tube defect including MMC who were admitted to the Cincinnati Children’s NICU after birth were included in the study. Infants without CIIM on imaging and those with genetic, cardiac, or gastrointestinal anomalies expected to affect feeding were excluded. We excluded those without CIIM because we felt that absence of CIIM possibly indicates a milder phenotype, and on fetal imaging often reflects that the lesion is actually closed or skin covered. Fetal and neonatal deaths and infants who transferred hospitals before discharge were also excluded due to the primary outcome not being available. Data was collected retrospectively from admission, at discharge, and at 3, 6, and 12 months corrected gestational age (CGA). This study was approved by the Institutional Review Board.

### Definition of outcomes

The primary outcome was a need for feeding tube support (nasogastric (NG) or gastric (GT)) at initial hospital discharge. Additional components of the primary outcome included primary feeding route at discharge, defined as the method by which >50% of enteral intake was achieved, and reason for feeding tube. These outcomes were again assessed at 3, 6, and 12 months CGA to evaluate longitudinal need for feeding tube.

As secondary outcomes, we evaluated time and route of first and full *per os* (PO) and any enteral feeds, rates of direct breastfeeding (DBF) and receiving mother’s own milk (MOM), number of days NPO, and video swallow study results. Non-feeding characteristics and outcomes included hospital length of stay, days on positive pressure and mechanical ventilation, days in positioning restrictions, episodes of infection, documented STS contact, weight z-scores, postnatal imaging results, need for tracheostomy or other respiratory support, and complications of prematurity.

### Definitions

A previously described grading scale was used to determine degree of CIIM as follows: Grade 1 had no cerebellar ectopia and downward sloping tentorium with a patent fourth ventricle and cisterna magna; grade 2 had cerebellar ectopia with effacement of the fourth ventricle but a patent cisterna magna; grade 3 had cerebellar ectopia and effacement of both the fourth ventricle and cisterna magna [8, 9]. This grading was performed on initial fetal MRI. Presence or absence of HH was also determined based on initial fetal MRI, and reversal of HH was determined by comparing initial fetal MRI and postnatal MRI. Infants born at 37 weeks or greater were considered term. Full feeds were defined as 120–140 ml/kg/day, or for older gestational ages, appropriate intake for age. Positive pressure ventilation was defined as continuous positive airway pressure (CPAP), non-invasive positive pressure ventilation (NIPPV), or high-flow nasal cannula (HFNC) > 2 L. NICHD criteria was used to determine bronchopulmonary dysplasia (BPD) diagnosis. Bell’s staging was used for necrotizing enterocolitis (NEC) classification. Diagnosis of clinical infection was based on antibiotic treatment greater than 48 hours.

### Statistical analysis

Descriptive statistics were used including percentages for categorical variables and means and standard deviations, or medians and IQR, for continuous variables. Wilcoxon rank sum test was used to compare group differences for numerical/ordinal variables to be robust against non-normal distribution and unequal variance assumptions. Chi-square or Fisher’s exact test was used for associations between nominal variable and group. Logistic regression was used to investigate the relationship between binary feeding tube outcome at discharge with the repair group variable. Generalized estimating equation (GEE) was used to analyze the longitudinal binary feeding tube outcome to account for the correlation within individuals across repeated measures. Interaction term between time and repair group was tested and kept in the model if significant. Both regression models adjusted for potential confounders that included GA at birth, ventriculomegaly on prenatal ultrasound, and intervention for hydrocephalus in the first year of life. To visually present the longitudinal trajectory of probability of having feeding tube by repair groups, generalized additive mixed effect model (GAMM) was used, where subjects were treated as random effect. SAS 9.4 was used for analysis and R4.0.4 (13) and R packages “mgcv” 1.8-17 were used for GAMM. Statistical significance was defined as a two-sided P value less than 0.05. No multiple testing adjustments were made.

## Results

### Patient population

**Fig. 1:**
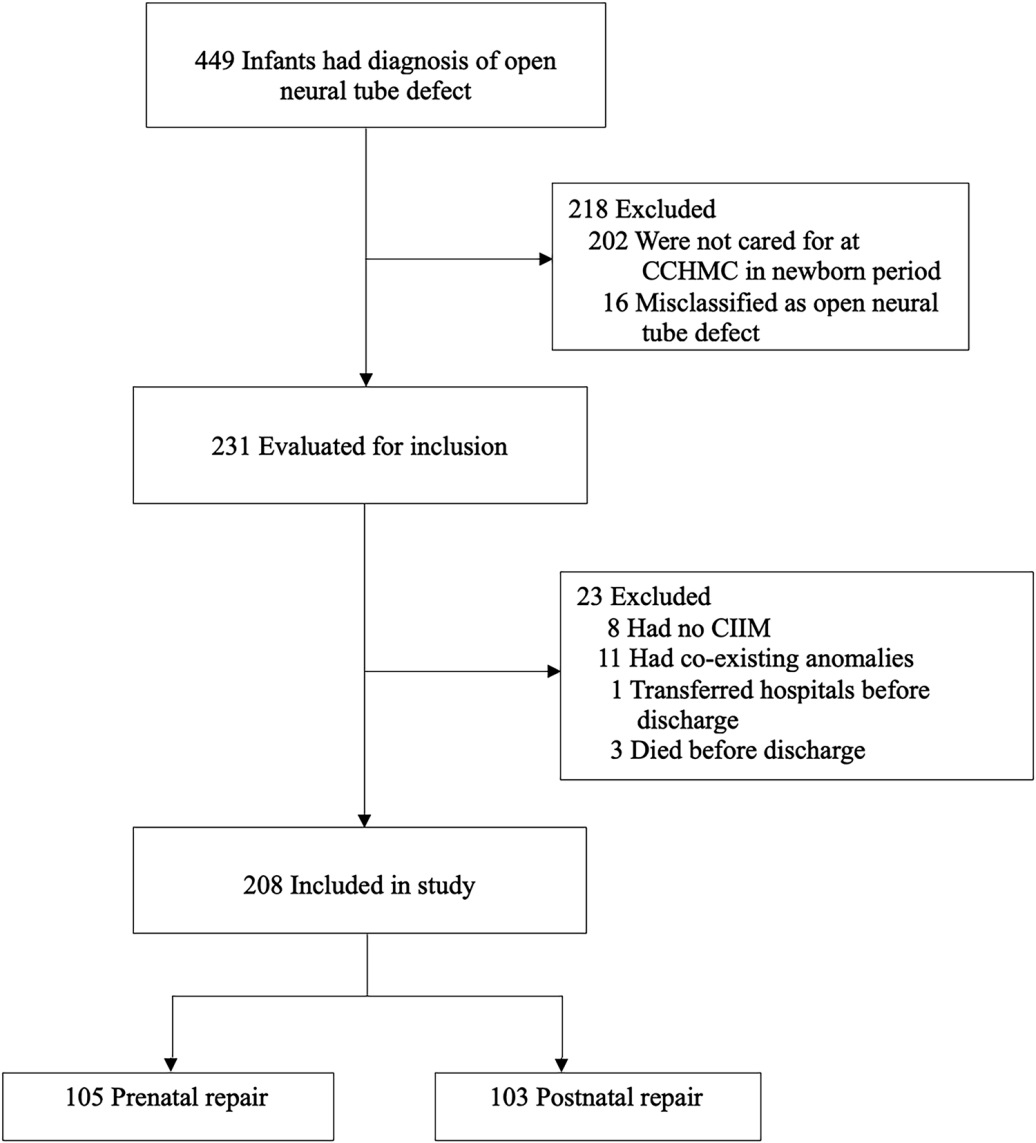
Patient inclusion and exclusion.

Two hundred and thirty-one infants with MMC were born during the study period. Twenty-three infants were excluded: eight with no CIIM, eleven with genetic, cardiac, or gastrointestinal anomalies expected to affect feeding, three died before discharge, and one transferred hospitals before discharge. Two hundred and eight infants met inclusion criteria, 105 repaired prenatally and 103 repaired postnatally (Fig. 1).

Table 1 demonstrates the relevant maternal characteristics. Age, marital status, race/ethnicity, and comorbidities such as diabetes and chronic hypertension were similar between groups. Of note, there was a higher baseline BMI in the postnatal repair group (31.9 vs 28.5, *p* = 0.002). In the postnatal repair group, 22% were candidates for prenatal repair but elected not to pursue this option. The remainder did not qualify for prenatal repair, mainly due to late diagnosis, maternal BMI, and other maternal conditions. In the prenatal repair group, there were nearly equal numbers of open and fetoscopic repair; these were performed at an average of 24.7 weeks. Mothers who underwent prenatal repair had higher rates of obstetric complications, especially preterm premature rupture of membranes and preterm labor. On prenatal ultrasound, the postnatal repair group had more thoracic and sacral lesions and larger degree of ventriculomegaly. There were equal rates of severe, grade 3 CIIM between both groups.

**Table 1:** Maternal and Prenatal Characteristics^a^.

	Prenatal Repair *N* = 105	Postnatal Repair *N* = 103	*P* Value
Race/Ethnicity			0.8413
White	99 (94.3)	96/102 (94.1)	
Hispanic	3 (2.9)	4/102 (3.9)	
African American	3 (2.9)	2/102 (2.0)	
Age at initial evaluation (years) - mean ± SD	28.4 ± 5.5	27.4 ± 5.6	0.2023
Marital Status			0.0641
Married	90 (85.7)	78/100 (78.0)	
Partnered	12 (11.4)	10/100 (10.0)	
Single	2 (1.9)	10/100 (10.0)	
Separated	1 (1.0)	2/100 (2.0)	
BMI at initial evaluation	28.5 ± 4.6	31.9 ± 7.3	0.0022
Diabetes			0.2289
Gestational	10 (9.5)	14/102 (13.7)	
Pre-gestational	0 (0.0)	2/102 (2.0)	
Hypertension	13 (12.4)	18/102 (17.6)	0.2884
Medication/Substance Use^b^	17 (16.2)	13 (12.7)	0.4814
Candidate for Repair	105 (100)	22/99 (22.2)	<0.0001
Reason for not being a repair candidate			
Maternal BMI > 40		20/77 (26.0)	
Late gestational age		16/77 (20.1)	
No HH		6/77 (7.8)	
Sacral lesion		9/77 (11.7)	
Maternal risk factor		19/77 (24.7)	
Other		18/77 (23.4)	
Prenatal repair type			
Open	52 (49.5)		
Fetoscopic	53 (50.5)		
GA at repair - mean ± SD	24.7 ± 0.7		
Obstetric complications^c^	90 (86.5)	16 (16.2)	<0.0001
Delivery mode			0.0094
Vaginal	27 (25.7)	12 (11.7)	
C-Section	78 (74.3)	91 (88.3)	
**Imaging Findings**			
Lesion level on ultrasound			0.0145
Thoracic	1 (1.0)	6/101 (5.9)	
L1-L2	17 (16.2)	21/101 (20.8)	
L3-L4	48 (45.7)	41/101 (40.6)	
L5-S1	36 (34.3)	22/101 (21.8)	
Sacrum	3 (2.9)	11/101 (10.9)	
Ventricle size (mm) on ultrasound at time of initial evaluation - mean ± SD	11.5 ± 3.6	13.3 ± 4.4	0.0041
Severity of ventriculomegaly			0.0817
None (<10 mm)	39/103 (37.9)	24/99 (24.2)	
Mild (10-12 mm)	23/103 (22.3)	19/99 (19.2)	
Moderate (13-15 mm)	25/103 (24.3)	30/99 (30.3)	
Severe (>15 mm)	16/103 (15.5)	26/99 (26.3)	
HH on pre-operative fetal MRI	105 (100.0)	88/95 (92.6)	0.0048
Chiari grade based on fetal MRI			0.0622
1	0 (0.0)	5/95 (5.3)	
2	13 (12.4)	11/95 (11.6)	
3	92 (87.6)	79/95 (83.2)	

^a^Unless otherwise noted, data are presented as *n*(%).

^b^SSRI, opiate, THC, and/or tobacco.

^c^PPROM, PTL, chorio-amniotic separation, placental abruption, pre-eclampsia and/or amniotic bands.

### Neonatal characteristics and comorbidities

Table 2 depicts neonatal characteristics and comorbidities. Mean gestational age (GA) and birthweight were lower in the prenatal repair group (33w vs 37.7w, *p* = <0.0001; 2111 vs 3229 grams, *p* = <0.001). Prenatally repaired infants were less likely to require intervention for hydrocephalus in the first year of life (34% vs 87.4%, *p* = <0.0001). Of those who did, 22.9% occurred during NICU admission, compared to 87.8% in the postnatal repair group (*p* = <0.0001). Seventy nine percent in the prenatal repair group had reversal of HH postnatally, compared to 4.5% in the postnatal repair group (*p* = <0.0001). Infants undergoing postnatal repair were more likely to have shunt complications and complications related to MMC repair. There were no differences in number of days in positioning restrictions related to MMC wound healing.

**Table 2:** Neonatal Characteristics and Comorbidities^a^.

	Prenatal Repair *N* = 105	Postnatal Repair *N* = 103	*P* Value
GA at birth (weeks) - mean ± SD	33.0 ± 3.4	37.7 ± 2.0	<0.0001
Birth weight (g) - mean ± SD	2110.7 ± 736.9	3229.0 ± 646.9	<0.0001
Male sex	55 (52.4)	53 (51.5)	0.8938
Intervention for hydrocephalus in first year of life	35/103 (34.0)	90 (87.4)	<0.0001
VPS	33/35 (94.3)	89/90 (87.4)	0.1891
EVD	1/35 (2.9)	15/90 (16.7)	0.0398
Other	3/35 (8.6)	2/90 (2.2)	0.1332
Intervention for hydrocephalus before NICU discharge	8/35 (22.9)	79/90 (87.8)	<0.0001
Reversal of HH postnatally	83 (79.0)	4/88 (4.5)	<0.0001
Complications of shunt^b^	7 (6.7)	33 (32.4)	<0.0001
Complications of repair^c^	16 (15.4)	32 (31.1)	0.0075
Days in positioning restrictions – median (IQR)	10.0 (4.0, 23.0)	13.0 (7.0, 19.0)	0.1054
Length of hospitalization, days	31.0 (16.0, 56.0)	20.0 (16.0, 30.0)	0.0167
CGA at discharge - mean ± SD	38.6 ± 2.4	42.8 ± 9.4	0.0005
Days on mechanical ventilation - mean ± SD	2.1 ± 13.0	10.2 ± 41.6	<0.0001
Days on positive pressure ventilation - mean ± SD	7.4 ± 14.2	2.9 ± 11.2	<0.0001
Tracheostomy in first year of life	0 (0.0)	6 (5.8)	0.0137
Severe BPD	6 (5.7)	2 (1.9)	0.2796
Respiratory support at discharge due to BPD	8 (7.6)	2 (1.9)	0.1010
Any NEC	3 (2.9)	2 (1.9)	1.0000
Surgical NEC	0 (0.0)	1 (0.9)	1.0000
Neurologic injury (Grade 3 or 4 IVH; PVL)	3 (2.9)	0 (0.0)	0.2464
Number of infection episodes - mean ± SD	1.1 ± 0.3	2.3 ± 2.2	0.0115
Infection type			
Culture proven	14/20 (70.0)	9/11 (81.8)	0.6757
Culture negative	6/20 (30.0)	3/11 (27.3)	1.0000
Infection Source			
Blood	1/20 (5.0)	2/11 (18.2)	0.2814
Urine	14/20 (70.0)	6/11 (54.5)	0.4524
Pneumonia	1/20 (5.0)	4/11 (36.4)	0.0416
Meningitis	0/20 (0.0)	1/11 (9.1)	0.3548
Other	1/20 (5.0)	1/11 (9.1)	1.0000
Days with central line – median (IQR)	0.0 (0.0, 9.0)	0.0 (0.0, 8.0)	0.2695

^a^Unless otherwise noted, data are presented as *n*(%).

^b^Shunt malfunction, infection and/or revision.

^c^Dehiscence, wound infection and/or return to OR/revision.

The prenatal repair group had a longer median length of hospitalization (31 days vs 20 days, *p* = 0.0167) and higher mean days on positive pressure ventilation, while the postnatal repair group had higher mean days on mechanical ventilation. Several infants in the postnatal repair group underwent tracheostomy in the first year of life, which was not seen in those undergoing prenatal repair (5.8% vs 0%, *p* = 0.0137). There was a significant burden of infection in both groups, but a higher number of episodes in the group undergoing postnatal repair. There were no statistical differences in the low rates of other important comorbidities including severe BPD, NEC, neurologic injury including severe intraventricular hemorrhage (IVH) and periventricular leukomalacia (PVL), and days with a central line.

### Primary outcome

The primary outcome was a need for feeding tube support (NG or GT) at initial hospital discharge, for which there was no difference between groups (Table 3). 9.5% of infants in the prenatal repair group and 13.6% in the postnatal repair group were discharged with a feeding tube (*p* = 0.3585; adjusted OR 0.759 [95% CI 0.174, 3.308]). Primary feeding route was oral in 98% of infants repaired prenatally and 90% repaired postnatally (*p* = 0.0467). There were some differences in reason for feeding tube support, though not significant: 20% in the prenatal repair group and 50% in the postnatal repair group required their feeding tube due to aspiration (*p* = 0.2099). The majority in the prenatal repair group required their feeding tube due to inadequate PO intake (80% vs. 42.9%, *p* = 0.1041) (Table 3).

**Table 3:** Neonatal Outcomes^a^.

	Prenatal Repair *N* = 105	Postnatal Repair *N* = 103	*P* Value	Adjusted OR (95% CI)^b^
Feeding tube at discharge	10 (9.5)	14 (13.6)	0.3585	0.759 (0.174, 3.308)
Primary feeding route at discharge			0.0467	
Oral	103 (98.1)	93 (90.3)		
NG	1 (1.0)	4 (3.9)		
GT	1 (1.0)	6 (5.8)		
Reason for feeding tube				
Aspiration	2/10 (20.0)	7/14 (50.0)	0.2099	
Inadequate PO intake	8/10 (80.0)	6/14 (42.9)	0.1041	
Respiratory support	0/10 (0.0)	3/14 (21.4)	0.2391	
Feeding tube at 3mo CGA	2/101 (2.0)	11/100 (11.0)	0.0093	
Primary feeding method at 3mo CGA			0.0720	
Oral	99/101 (98.0)	89/100 (89.0)		
NG	1/101 (1.0)	3/100 (3.0)		
GT	1/101 (1.0)	5/100 (5.0)		
Transpyloric	0/101 (0.0)	3/100 (3.0)		
Feeding tube at 6mo CGA	2/101 (2.0)	8/98 (8.2)	0.0559	
Primary feeding method at 6mo CGA			0.0780	
Oral	98/100 (98.0)	90/98 (91.8)		
NG	1/100 (1.0)	1/98 (1.0)		
GT	1/100 (1.0)	6/98 (6.1)		
Transpyloric	0/100 (0.0)	1/98 (1.0)		
Feeding tube at 12mo CGA	1/99 (1.0)	7/95 (7.4)	0.0323	
Primary feeding method at 12mo CGA			0.0364	
Oral	98/99 (99.0)	88/95 (92.6)		
GT	1/99 (1.0)	6/95 (6.3)		
Transpyloric	0/99 (0.0)	1/95 (1.1)		
Need for feeding tube at 53w CGA			0.0250	0.325 (0.121, 0.872)
Feed tube at any point during admission	87 (82.9)	68 (66.0)	0.0053	
DOL first enteral feed – median (IQR)	1.0 (0.0, 1.0)	2.0 (1.0, 2.0)	<0.0001	
Feeding route at first enteral feed			<0.0001	
Oral	28 (26.7)	71 (68.9)		
NG/OG	62 (59.0)	30 (29.1)		
DBF	12 (11.4)	1 (1.0)		
Oral+NG/OG	2 (2.9)	1 (1.0)		
DOL full enteral feeds – median (IQR)	6.0 (4.0, 11.0)	6.0 (5.0, 8.0)	0.6600	
DOL first PO feed – median (IQR)	3.0 (1.0, 24.0)	2.0 (1.0, 3.0)	0.0392	
DOL full PO feeds – median (IQR)	24.0 (10.0, 48.0)	7.0 (5.0, 11.0)	<0.0001	
CGA at full PO feeds (if born <37w) - mean ± SD	37.3 ± 2.0	38.6 ± 2.2	0.0049	
Number of days NPO - median (IQR)	0.0 (0.0, 1.0)	2.0 (1.0, 3.0)	<0.0001	
Video swallow study during admission	18 (17.1)	22 (21.4)	0.4404	
Video swallow study results			0.0710	
Normal	5/18 (27.8)	1/22 (4.5)		
Aspiration	5/18 (27.8)	13/22 (59.1)		
Other abnormalities	8/18 (44.4)	8/22 (36.4)		
Attempted DBF during admission	81 (77.1)	60 (58.3)	0.0036	
DOL first DBF – median (IQR)	6.0 (1.0, 19.0)	7.5 (5.0, 14.0)	0.5699	
DBF at discharge	55 (52.4)	38 (36.9)	0.0247	
MOM at discharge	88 (83.8)	68 (66.0)	0.0031	
STS during admission	80 (76.2)	37 (35.9)	<0.0001	
DOL first STS – median (IQR)	4.5 (1.0, 8.5)	7.0 (4.0, 14.5)	0.0491	
Weight z-scores – median (IQR)				
Birth	0.2 (-0.2, 0.6)	0.3 (-0.5, 0.8)	0.8357	
Discharge	-0.5 (-1.5, -0.1)	-0.3 (-0.9, 0.5)	0.0015	
3mo CGA	-0.5 (-1.5, 0.4)	0.0 (-0.9, 0.8)	0.0030	
6mo CGA	0.2 (-0.7, 0.9)	0.5 (-0.4, 1.2)	0.1069	
12mo CGA	0.4 (-0.6, 1.4)	0.5 (-0.5, 1.1)	0.9336	

^a^Unless otherwise noted, data are presented as *n*(%).

^b^GEE for longitudinal feeding tube outcome adjusting for GA at birth, ventriculomegaly on prenatal ultrasound, and intervention for hydrocephalus in the first year of life and interaction terms if significant.

**Fig. 2:**
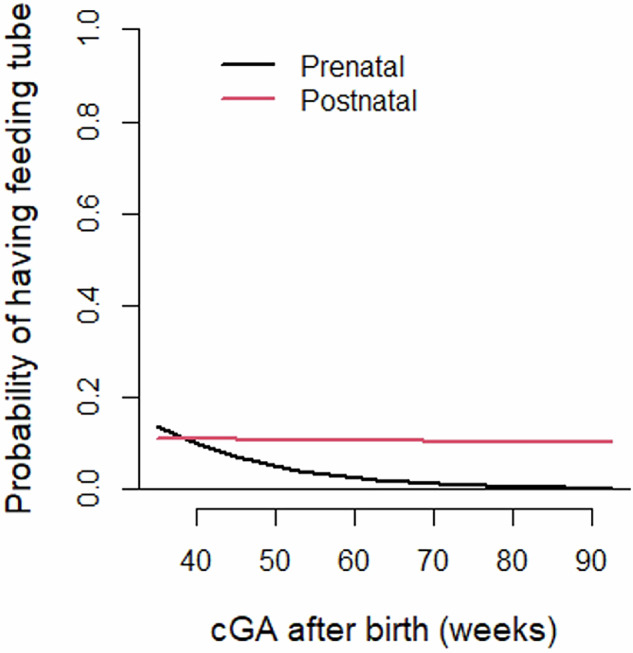
Longitudinal probability of need for feeding tube beginning at term corrected age. Prenatal vs. Postnatal need for feeding tube at 53 weeks CGA: Adjusted OR 0.325 [95% CI 0.121, 0.872], *p* = 0.025.

The need for feeding tube decreased over the first year of life in both groups but was more pronounced in the prenatal repair group. By 53 weeks CGA, the prenatal repair group had decreased odds of requiring a feeding tube (OR 0.325 [95% CI 0.121, 0.872], *p* = 0.025) when adjusting for GA at birth, degree of ventriculomegaly on prenatal ultrasound, and intervention for hydrocephalus in the first year of life (Fig. 2). By 12 months CGA, 1% in the prenatal repair group and 7.4% in the postnatal repair group still required a feeding tube (*p* = 0.0323), and all of these infants relied on a GT or gastrojejunostomy (GJ) tube for 100% of their feeds (Table 3).

### Secondary outcomes

Secondary outcomes are depicted in Table 3. The prenatal repair group was more likely to have a feeding tube during admission (82.9% vs. 66.0%, *p* = 0.0053) and had longer time to first and full oral feeds. 68.9% in the postnatal repair group received their first enteral feed orally, compared to 26.7% in the prenatal repair group (*p* = <0.0001). The postnatal repair group had longer time to first enteral feed and more NPO days on average.

Eighteen infants in the prenatal repair group and 22 in the postnatal repair group had a video swallow study in the first year of life. Of these, aspiration was present in 27.8% of infants repaired prenatally and 59.1% of infants repaired postnatally (*p* = 0.0710). The prenatal repair group had higher rates of receiving any MOM at discharge, attempting DBF during admission, DBF at discharge, and having documented STS contact during admission.

The prenatal repair group had lower z-score for weight at discharge and 3 months CGA, but there was no difference in weight z-scores at birth, term corrected, 6 months and 12 months CGA.

## Discussion

Compared to standard postnatal MMC repair, prenatal repair was associated with equal rates of feeding tube use at hospital discharge, but lower need for feeding tube use over the first year of life and no negative effects on growth during the follow up period. There was also a lower need for tracheostomy in the first year of life. These infants also had significantly higher rates of HH reversal following prenatal repair. These data suggest that prenatal repair is associated with decreased severe symptomatic CIIM and brainstem dysfunction. Of great importance to families, infants repaired prenatally were also more likely to experience bonding interactions including breastfeeding and STS contact during NICU admission. While it is well established that prenatal MMC repair improves motor function, decreases the need for VP shunt, and reverses HH, few studies have evaluated effects on symptoms of brainstem dysfunction, and to our knowledge this is the first study directly comparing feeding outcomes between repair types.

Feeding is a challenging problem in the population undergoing fetal surgery, as it can be affected by brainstem dysfunction as well as prematurity. While the primary outcome of feeding tube use at hospital discharge did not differ between groups, we believe that our center’s strong follow up program and Remote Patient Monitoring structure facilitates early and aggressive discharge practices with temporary feeding tubes in place. With an increase in the number of prenatal repair cases over time, this may have hidden an earlier detection in differences between groups as more infants were discharged with feeding tubes. Additionally, while not statistically significant, the prenatal repair group was more likely to require feeding support at discharge due to inadequate oral intake, unlike the postnatal repair group who was more likely to have aspiration. Moreover, the prenatal repair group had a more pronounced decrease in the need for feeding tube after term corrected age compared to the postnatal repair group and had decreased odds of requiring a feeding tube by 53 weeks CGA. This suggests that need for feeding tube in the prenatal repair group was likely related to developmental immaturity which improved over time, while the postnatal repair group was affected by CIIM-related swallowing dysfunction and aspiration which are less likely to resolve. Hydrocephalus and presence of a VP shunt, which were more prevalent in the postnatal repair group, can also have negative effects on feeding, but these were adjusted for in our analysis. No infants repaired prenatally required a tracheostomy, further suggesting that prenatal repair may decrease the risk of developing severe symptoms of brainstem dysfunction. At our center, the performance of video swallow studies is done based on symptoms or care team concerns regarding swallow safety rather than as a routine screening tool. The frequency of aspiration in our cohort was approximately 60% with postnatal repair when a video swallow study was performed, yet only 13% overall, which is consistent with other studies evaluating symptomatic CIIM in this population [15, 21].

Postnatally repaired infants in our study were less likely to experience bonding interactions including breastfeeding and STS contact. The reason for this is unclear but could potentially be due to barriers such as longer time spent NPO and requiring mechanical ventilation, as well as family stress or discomfort around holding an infant with a recent surgical repair, particularly those with difficult wound healing. Known benefits of breastfeeding and STS including improved wound healing, decreased risk of infection, and maternal-infant bonding are important for infants repaired postnatally, and it is necessary to further investigate this discrepancy between repair groups. This may represent an area for quality improvement efforts, regardless of strategy of MMC repair.

While the goal of the study was to assess feeding outcomes in infants with MMC, we would be remiss not to comment on the other important differences between the groups. While there was a high rate of preterm birth less than 37 weeks gestation, there was not a large burden of related comorbidities. This may reflect the fact that these infants are largely born in the moderate to late preterm window, when rates of these comorbidities are expected to be lower. This however does not minimize the short- and long-term issues faced by late preterm infants [22]. In addition to oral intake, these infants often require feeding tubes for growth. Our data show that the prenatal repair group initially had a higher drop in their weight z-score after birth compared to the postnatal repair group, but they exhibited catch up growth over time even as their NG tubes were removed. This supports our belief that feeding tube use in the prenatal repair group was largely due to developmental immaturity that improved over time. Rates of severe BPD were low, and no patients required tracheostomy for any reason in the prenatal repair group, a major improvement compared to those repaired postnatally.

Our study had several limitations. Due to the retrospective design, we were unable to control for all potential confounders. The relatively small sample size, particularly of infants with the primary outcome, likely affected the statistical power of the study. There were many patients identified in our EPIC query who had a diagnosis of MMC but did not meet inclusion criteria due to receiving care at our center outside of the study period and after the first year of life. We therefore do not have early feeding data on these patients, which would have allowed us to strengthen the statistical power by increasing sample size. Given the long study period, there were likely practice changes over time that may have affected outcomes, for example, quality initiatives to improve rates of breastfeeding and STS contact in all infants. There are several other CIIM-related feeding difficulties that we did not evaluate such as long feeding times, choking, and reflux, which can be difficult to illicit via chart review. However, we feel that our outcomes of feeding tube use and aspiration encompass the most clinically significant feeding manifestations of CIIM. Lastly, as a single site study our results may not be generalizable to other centers as practices for supplemental feeding vary largely. Despite this, we view the single site design as a strength of our study as our follow up program allows us to standardize the variable of supplemental feeding practices. While our center does not have a standard protocol for discontinuation of supplementation, it is our unit standard practice that for infants who require feeding tubes due to inadequate oral volumes related to immature feeding skills, NG tubes are generally removed when an infant takes 80 percent or greater of feeds orally for two consecutive days. Infants discharged with a feeding tube or caloric supplementation are monitored via our Remote Patient Monitoring program. Decision to discontinue supplementation is based on a combination of weight gain and progression of oral feeding volumes.

In conclusion, our study found that prenatal MMC repair was associated with lower need for long-term feeding support as well as lower need for tracheostomy, suggesting that prenatal repair may reduce symptomatic CIIM and brainstem dysfunction. This study provides new information regarding benefits of prenatal MMC repair and potential clinical changes related to reversal of HH. In addition to aiding in prenatal counseling regarding the known risks and benefits of fetal surgery, these findings can help physicians determine the likely trajectory of an infant’s feeding tube use and guide decisions about surgical GT placement. A larger, multi-site study would provide power to these conclusions and may be pursued amongst sites with similar fetal intervention populations.

## Data Availability

Data will be made available upon reasonable written request to the corresponding author.
